# Supplementary material for: In-Hospital Patient Education Markedly Reduces Alcohol Consumption after Alcohol-Induced Acute Pancreatitis
Source: Nutrients. 2022 May 20;14(10):2131. doi: 10.3390/nu14102131 (PMC9144493; doi:10.3390/nu14102131)
Supplement: Supplementary file 1 [file nutrients-14-02131-s001.zip › nutrients-1710011-supplementary.pdf]

Table S1. Participating centres.

Centres	Patient number	%
Department of Translational Medicine, University of Pécs	73	73.74
Department of Gastroenterology, Szent György University Teaching Hospital of Fejér County	19	19.19
Department of Gastroenterology, University of Debrecen	7	7.07

Table S2. Data quality.

	N	Reported data	%
Age	99	99	100
Gender	99	99	100
Severity	99	97	98
Length of hospitalization	99	99	100
Previous RAP	99	99	100
Chronic pancreatitis	99	99	100
Hypertriglyceridaemia	99	88	89
Alcohol consumption (frequency)	99	99	100
Alcohol consumption (amount)	99	95	96
Admission GGT level	99	99	100
Discharge GGT level	99	93	94
1-month GGT level	99	99	100
Admission MCV level	99	99	100
Discharge MCV level	99	94	95
1-month MCV level	99	99	100
Self-reporting	99	95	96
**Total**	**99**	**97**	**98**

GGT – gamma-glutamyltransferase; MCV – mean corpuscular volume.

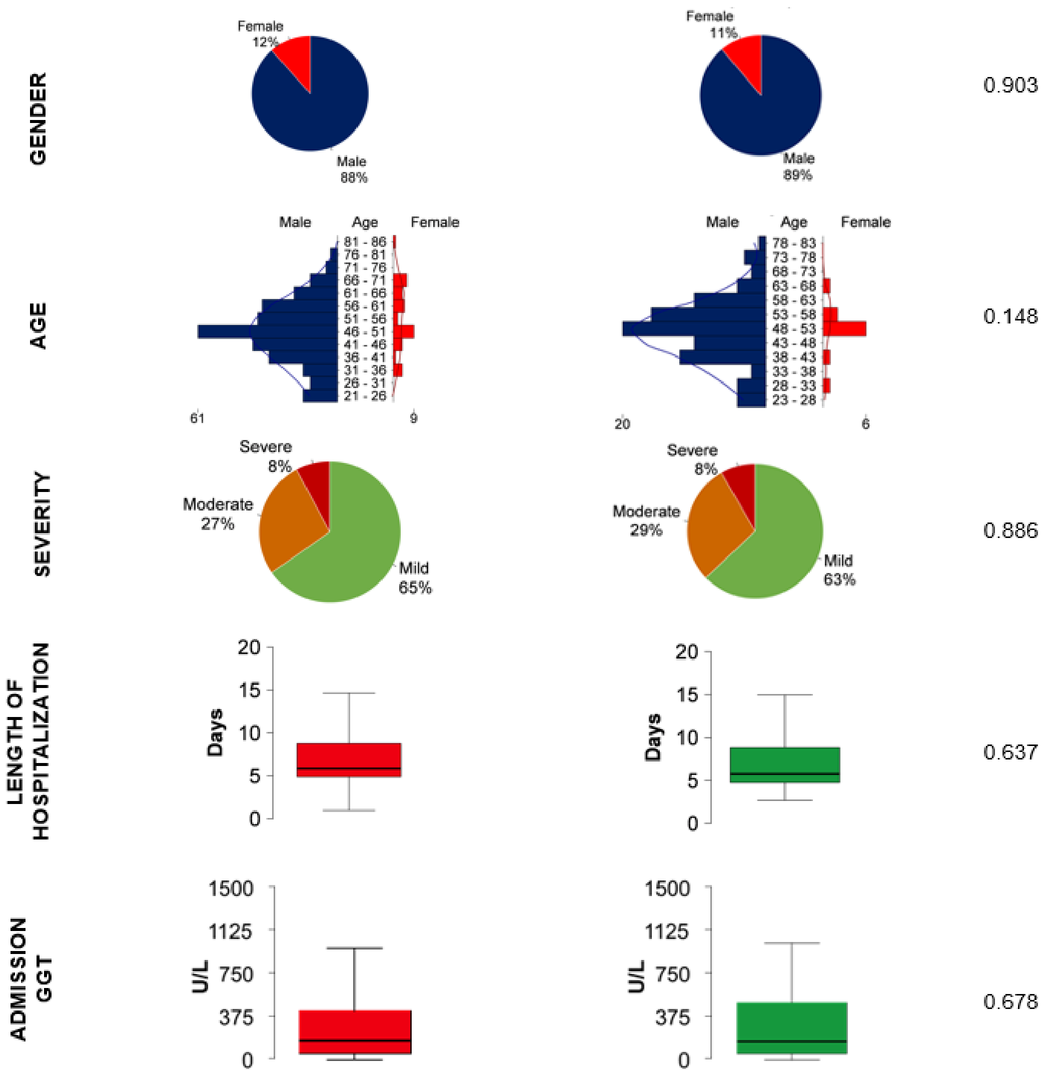

Figure S1. Representiveness. Comparison of basic characteristics (age, gender, severity, length of hospitalization, admission GGT level) of all patients with alcohol-induced acute pancreatitis and enrolled patients. There is no significant difference in terms of basic characteristics between the two groups.
